# Medical countermeasures for radiation induced health effects: report of an Interagency Panel Session held at the NASA Human Research Program Investigator’s Workshop, 26 January 2017

**DOI:** 10.1080/09553002.2019.1665214

**Published:** 2019-09-20

**Authors:** Lisa S. Carnell, Mary Homer, Keith Hoots, Heather Meeks, Pataje G. S. Prasanna, Carmen Rios, Lisa C. Simonsen, Lanyn P. Taliaferro, Lynne K. Wathen

**Affiliations:** a NASA Langley Research Center, Hampton, VA, USA; b US Department of Health and Human Services, Biomedical Advanced Research and Development Authority, Office of the Assistant Secretary for Preparedness and Response, Washington, DC, USA; c National Heart, Lung, and Blood Institute, National Institutes of Health, Bethesda, MD, USA; d Defense Threat Reduction Agency, Fort Belvoir, VA, USA; e US Department of Health and Human Services, Division of Cancer Treatment and Diagnosis, Radiation Research Program, National Cancer Institute, National Institutes of Health, Bethesda, MD, USA; f Division of Allergy, Immunology and Transplantation (DAIT), Radiation and Nuclear Countermeasures Program (RNCP), National Institute of Allergy and Infectious Diseases (NIAID), National Institutes of Health (NIH), Rockville, MD, USA

**Keywords:** Space, radiation, medical countermeasures, radioprotection, mitigator

## Abstract

An Interagency Panel Session organized by the NASA Human Research Program (HRP) Space Radiation Program Element (SRPE) was held during the NASA HRP Investigator’s Workshop (IWS) in Galveston, Texas on 26 January 2017 to identify complementary research areas that will advance the testing and development of medical countermeasures (MCMs) in support of radioprotection and radiation mitigation on the ground and in space. There were several areas of common interest identified among the various participating agencies. This report provides a summary of the topics discussed by each agency along with potential areas of intersection for mutual collaboration opportunities. Common goals induded repurposing of pharmaceuticals, nutraceuticals for use as radioprotectors and/or mitigators, low-dose/chronic exposure paradigms, late effects post-radiation exposure, mixed-field exposures of gamma-neutron, performance decrements, and methods to determine individual exposure levels.

## Introduction

NASA is preparing for the next frontier of exploration missions that will include sending astronauts to cis-lunar habitats, the moon and Mars, over the next 30 years. This requires NASA to understand the implications to the astronauts’ health with radiation being one of the greater unknowns. The International Space Station (ISS) has provided key evidence on the impact microgravity and living in space has on the human body; however, radiation exposures accumulated on the ISS are a fraction of what the astronauts will experience during longer, deep space missions. While shielding on spacecraft and in the habitats will provide some mitigation, it is impossible to prevent astronauts from being exposed to high-energy, low dose-rates of galactic cosmic radiation (GCR). To address the impact of radiation-induced health questions, NASA recently upgraded its Galactic Cosmic Ray Simulator to provide a more accurate representation of the space radiation environment in support of ground based research (Norbury et al. 2016). This facility will be critical when evaluating medical countermeasures (MCMs) to protect or mitigate radiation-induced health effects in astronauts exposed to GCR.

NASA has developed requirements and a plan to pursue MCMs to provide mitigation and reduce the overall radiation risk to astronauts (Carnell 2019). One aspect of NASA’s plan is to engage with interagency partners to leverage their existing research and development, to leam from them, and potentially expedite NASA’s goals. To accomplish this, it is necessary to understand the goals of each agency and identify common areas where collaborations can occur. This prompted the joint session organized by NASA Space Radiation Program Element (SRPE) during the NASA Human Research Program (HRP) Investigator’s Workshop (IWS).

**Figure 1.**
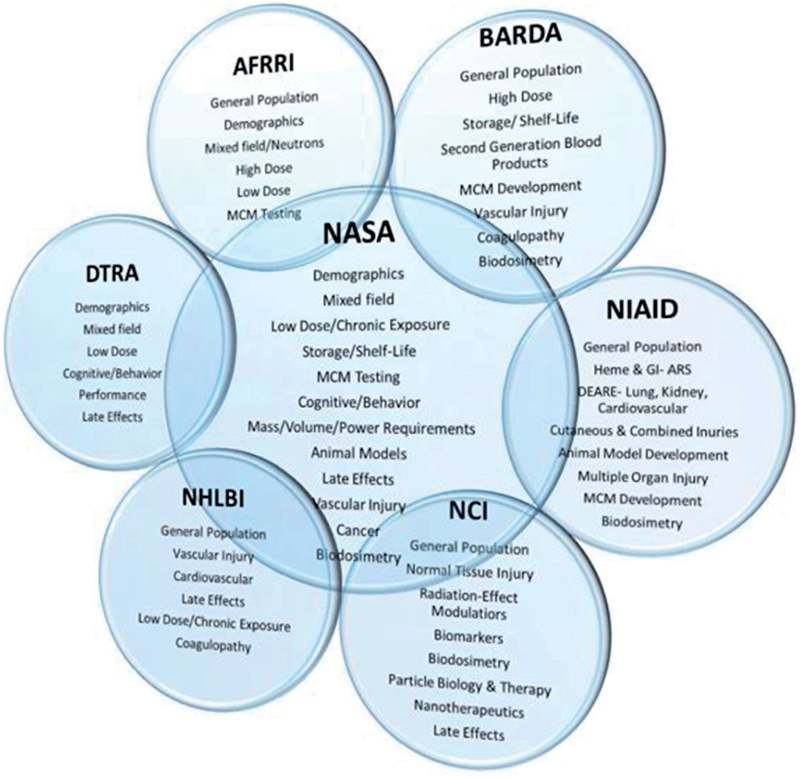
Agency areas of interest and their intersection with NASA.

The Interagency Panel Session was organized to address specific questions regarding radiation-induced health effects, exposure concerns, and MCM research and development of interest to each participating agency. It included presentations from several institutes under the National Institutes of Health (NIH) including the National Cancer Institute (NCI), National Institute for Allergy and Infectious Disease (NIAID), National Heart Lung and Blood Institute (NHLBI), Biomedical Advanced Research and Development Authority (BARDA), the Defense Threat Reduction Agency (DTRA), and NASA, along with attendance by the Armed Forces Radiobiology Research Institute (AFRRI). Each agency and institute identified unique requirements and goals for MCM development and implementation. Illustrated in Figure 1 (Carnell 2019) are the highlights for each agency and institute’s key areas of interest based on their requirements and goals.

Conventional medical intervention is associated with therapeutics delivered to ameliorate symptoms associated with specific indications. Currently, MCMs are under development to address the more complex attributes of radiation-induced health effects to support prevention, and reduction of toxicity and adverse health effects. These MCMs are classified as radioprotectors, developed to protect tissue prior to radiation exposure, and radiomitigators, intended to minimize the damage associated with radiation (Citrin et al. 2010).

Radioprotectors and radiomitigators have been in the spotlight for more than a decade post-9/11 era. The potential for a nuclear accident or worse, detonation, increased significantly and several efforts were born to develop ways to protect the public and military warfighter including, the NIH NIAID Radiation Nuclear Countermeasures Program (RNCP), and the BARDA. Their efforts have focused on the development of end-to-end solutions to respond to mass injuries associated with nuclear and radiological incidents. The primary goal has been rescuing victims from acute radiation exposures that may result in loss of life. Several agents have been developed, FDA approved, and stockpiled in a relatively short period to address these needs and many more are in the pipeline as potential candidates to include in the Strategic National Stockpile (SNS).

The military has concerns for the warfighter during these events and other activities that may expose troops to radiation. The possibility of performance decrements exists if troops are exposed to even relatively low doses (<2Gy) of radiation during missions or support efforts that may result in mission compromise (Brown et al. 1977). However, the negative effects of radiation exposure extend far beyond the potential for a nuclear disaster.

Millions of people treated annually with radiotherapy suffer from latent effects that disrupt their overall quality of life. The Radiation Research Program (RRP), Division of Cancer Treatment and Diagnosis under the NIH NCI is chartered with protecting normal tissue during radiation therapy and mitigating radiation-induced side effects. Latent effects from radiation exposure involve the vascular system to a great extent, which can compromise multiple organs in the body. The NIH NHLBI is interested in mitigating the effects on the vascular system post-radiotherapy. Understanding these health effects for terrestrial application also has benefit to NASA to address potential in-flight and latent effects anticipated post-long duration, deep space exploration missions.

### National Institutes of Health/National Cancer Institute

**Figure 2.**
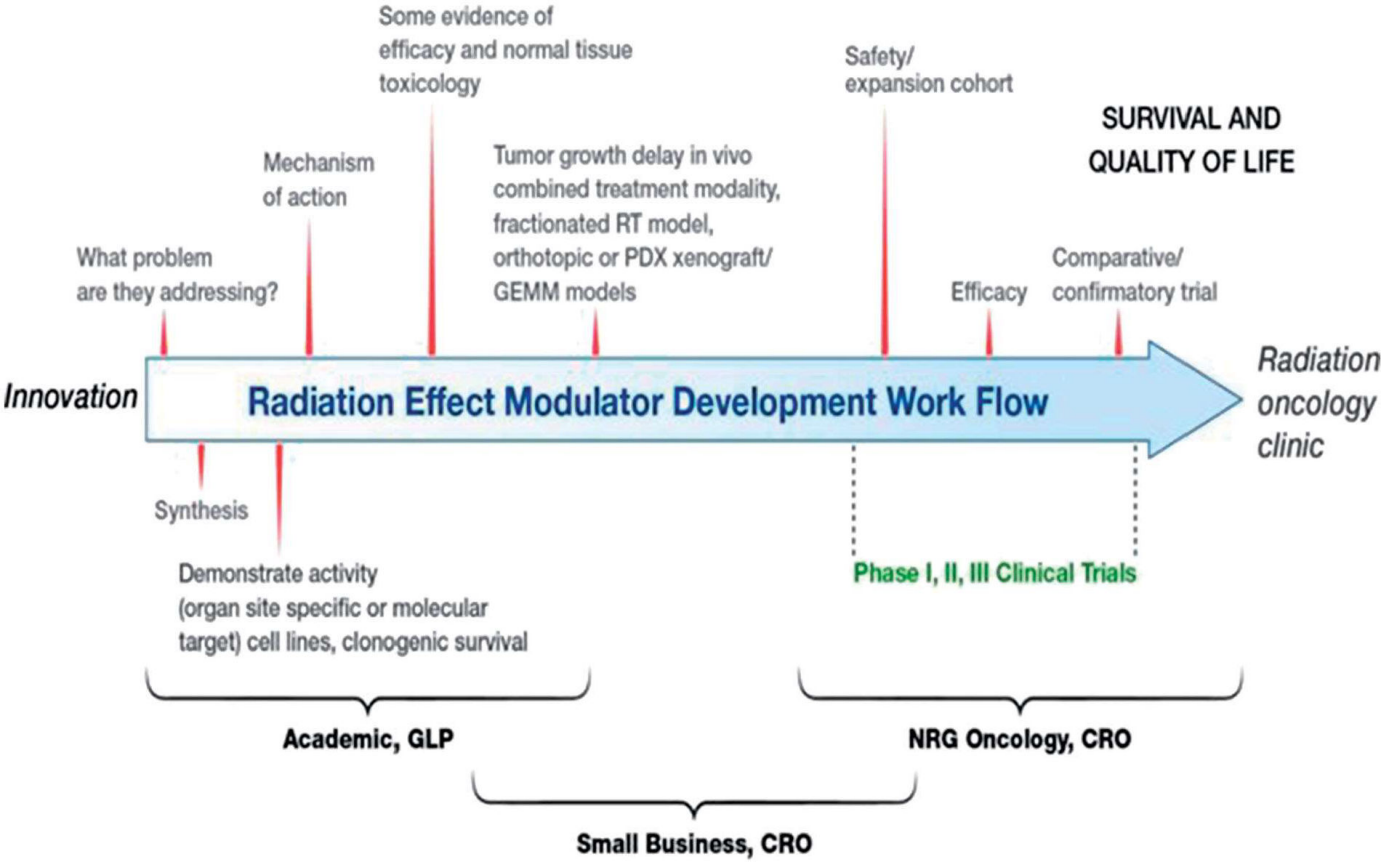
A suggested generalized workflow for the development of radioprotectors and mitigators for radiation oncology. The development of a radiation-effect modulator is a multi-step process from innovation to translation, as described in the illustration, which may involve the acquisition of intel lectual property from an academic or industry source for development and translation to radiation oncology clinic following regulatory approval. The various steps in this workflow may include the following: sourcing of intel lectual property, focusing development towards a solving a specific problem in the clinic, synthesis of the radiation-effect modulator, developing scientific evidence for organ/site-specific activity, evaluation of mechanism of action, formulation and dose/schedule optimizaron and performing studies on normal tissue toxicities, further evaluation in tumor bearing animals, if necessary, and conducting phase I, II, and III clinical trials. A dose interaction among academia, small businesses, and clinical trial workgroups is crucial for successful translation of a radiation-effect modulator for ultimate use In the clinic (Prasanna et al. 2015). © 2019 Radiation Research Society. GLP: good laboratory practice; CRO: Contract Research Organization. NRG - NSABP: National Surgical Adjuvant Breast and Bowel Project; RTOG: the Radiation Therapy Oncology Group; GOG: the Gynecologic Oncology Group.

Dr. Pataje Prasanna, RRP, Division of Cancer Treatment and Diagnosis under NIH/NCI (https://rrp.cancer.gov/default.htm), gave an overview of ‘Radioprotectors and Mitigators for Improving Radiotherapy’. Radiotherapy is currently used to treat half of all cancer patients and has become a curative modality. In 2012, there were 14.1 million new cancer cases and 7 million treated with radiotherapy. Projections for future cancer cases are staggering. By 2030, there will be an estimated 24.6 million new cáncer cases, and 12 million of those will be treated using radiotherapy (Yap et al. 2016). A focus for NCI is how to address post-treatment quality of life. Radiotherapy reduces cognitive fimetion in 50–90% of cancer patients treated for glioblastoma, and head and neck cancers (Greene-Schloesser and Robbins 2012). Radiation-induced brain injury involves inflammation, changes in the central nervous system (CNS) microenvironment, signaling dysfunction, vascular damage, injury to neurons and cellular organdies, demyelination, and collagen deposition (Greene-Schloesser et al. 2012; Balentova and Adamkov 2015). It was noted that apoptosis and necrosis appear to play a major role as well (Balentova and Adamkov 2015). Development of radioprotectors will allow for dose escalation with the goal of eliminating the tumor while a radiation mitigator will help improve post-treatment quality of life. Figure 2 (Prasanna et al. 2015) depiets the pathway for the translation of radiation effect modulators to the radiation oncology clinic (Prasanna et al. 2012, 2015; Citrin et al. 2017). The process involves moving the work through a logical hierarchy of model systems from in vitro based assays through in vivo tumor models and ultimately to the clinic. Early screening using in vitro systems could save resources and time.

### Department of Defense/Defense Threat Reduction Agency (DOD/DTRA)

The DTRA has two primary objectives: (1) to develop prophylaxes to prevent latent effects associated with radiation exposure that occurs during warfighter operations and (2) to develop environmental monitoring Solutions such as changes in water, soil, or biota for near- to mid-field (1–10 km from the site of interest) characterization of nuclear activity (DTRA 2019). DTRA’s approach for developing prophylaxes is to study intracellular response-recovery modes for different domains of life (bacteria, archaea, or eukarya), with a focus on understanding intrinsic radioresistance. Environmental monitoring surveillance approaches use -omics, genotypic, functional and phenotype changes related to exposure. Additional work in this area explores development of materials with multicatalytic centers for successive analyte characterization which increase signal veracity. Studies are designed to develop elements which can be incorporated into standard optical or electrochemical platforms. Other topics explore changes to local flora and fauna in the surrounding environment that are relatable to exposure of distinct Chemical species or level/type/quality of radiation. The demographics for military personnel, low-dose/low-dose rate and mixed neutron/gamma radiation field are complementary to NASA’s interests. DTRA is also concerned with performance decrements for the warfighter which complements NASA’s interest related to in-flight events that may occur with astronauts on long-duration missions.

### National Institutes of Health/National Institute of Allergy and Infectious Disease (NIAID)

**Figure 3.**
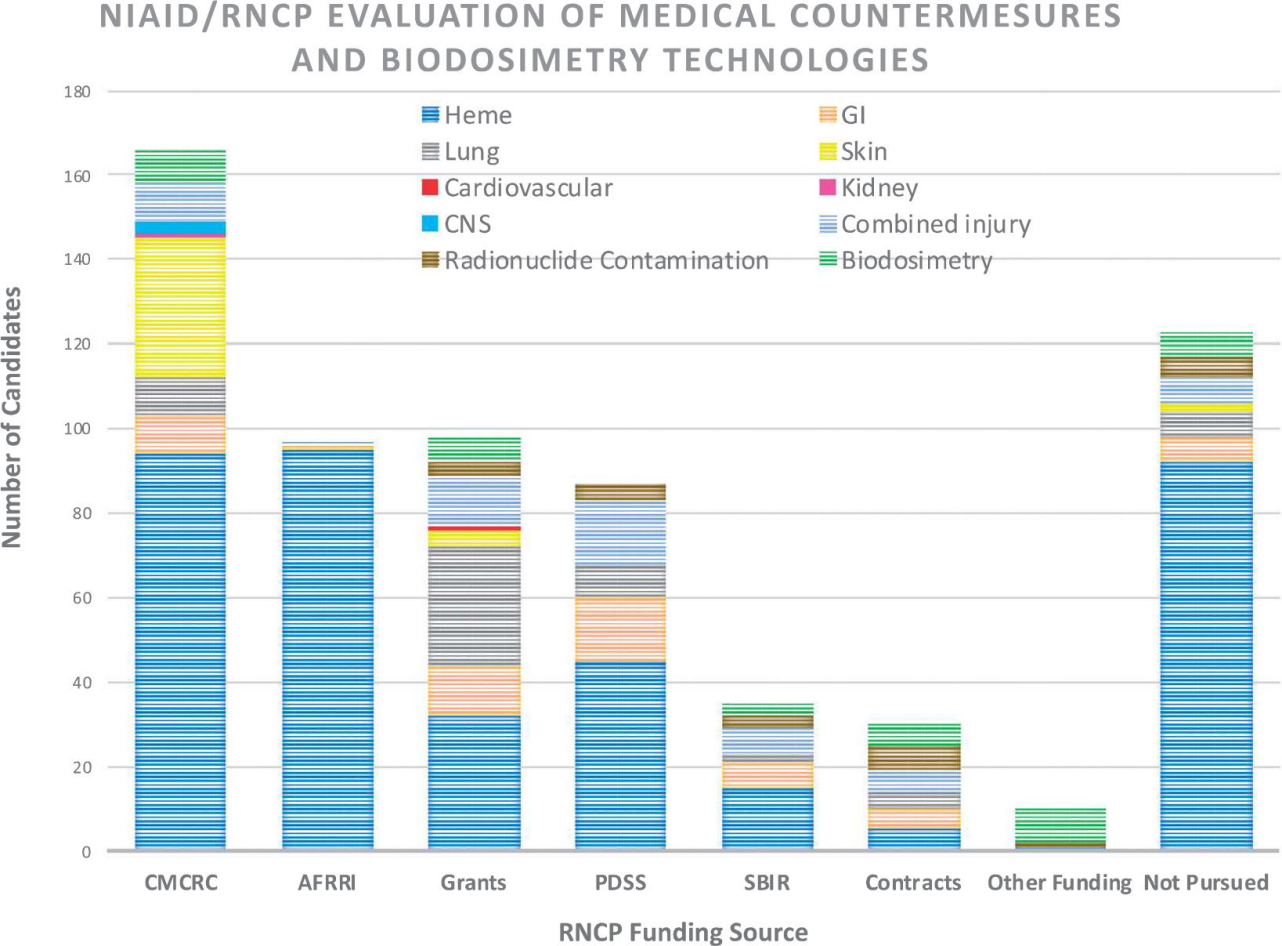
The NIAID/RNCP portfolio includes the evaluatlon of numerous MCMs and blodoslmetry technologies. Shown here are the number of MCM and biodosimetry candidates evaluated based on RNCP funding source or program area (CMCRC: Centers for Medical Countermeasures Against Radiation Consortium; AFRRI: Armed Forces Radiobiology Research Institute, program grants, PDSS: Product Development Support Services contract; SBIR: Small Business Innovation Research grants, program contracts and other funding mechanisms). Candidates that may not have met the criteria for evaluation or were too early for consideration are labeled ‘Not Pursued.’ In addition, the number of candidates evaluated or not evaluated is further broken down into scientific areas of interest. Heme: hematopoietic; Gl: gastrointestinal, lung, skin, cardiovascular, kidney; CNS: central nervous system, comblned injury, radionudide decontaminaron, biodosimetry.

NIAID Program Officers, Drs. Carmen Rios and Lanyn Taliaferro, provided background information on the RNCP. In 2004, NIAID was directed by the Department of Health and Human Services (HHS) to start a research program to accelerate development of radiation/nuclear MCMs for the SNS. NIAID’s primary mission is to support early to mid-stage research to develop radiation/nuclear MCMs and biodosimetry tools with an emphasis on three key areas: (1) drugs to treat or mitigate radiation injury 24hours post-exposure, (2) drugs to remove radioactive materials from the body and (3) biodosimetry tools and biomarker identifïcation to determine levels of radiation exposure as described in their strategic plan (NIH 2012). This is accomplished through grants, collaborative agreements, contracts, and interand intra-ageney agreements. Over 200 MCM candidates and biomarkers have been evaluated (Figure 3). Of these, six biodosimetry approaches which have reached higher technology readiness levels (TRLs) have transitioned to BARDA for advanced development, and two MCMs are in the DOD pipeline for prophylaxis development. NIAID’s efforts resulted in the fïrst two MCMs, Neupogen^®^ and Neulasta^®^, approved by the FDA under the Animal Rule with the indication to treat hematopoietic acute radiation syndrome (H-ARS) (FDA 2019). NI AID also received an FDA Investigational New Drug authorization to proceed with fïrst-in-human safety/PK evaluation of an oral radionuclide decorporation agent (hydroxypyridinone-3,4,3(l,2-HOPO)). Delayed effects from acute radiation exposure (DEARE) is another area of interest in NIAID’s portfolio. DEARE along with H-ARS mitigators are areas of common interest between NASA and NIAID. NASA is concerned with acute exposures from solar particle events (SPEs) and delayed effects from these exposures could impact quality of life for crew upon returning to Earth.

### Assistant Secretary for Preparedness and Response/Biomedical Advanced Research and Development Authority (ASPR/BARDA)

**Figure 4.**
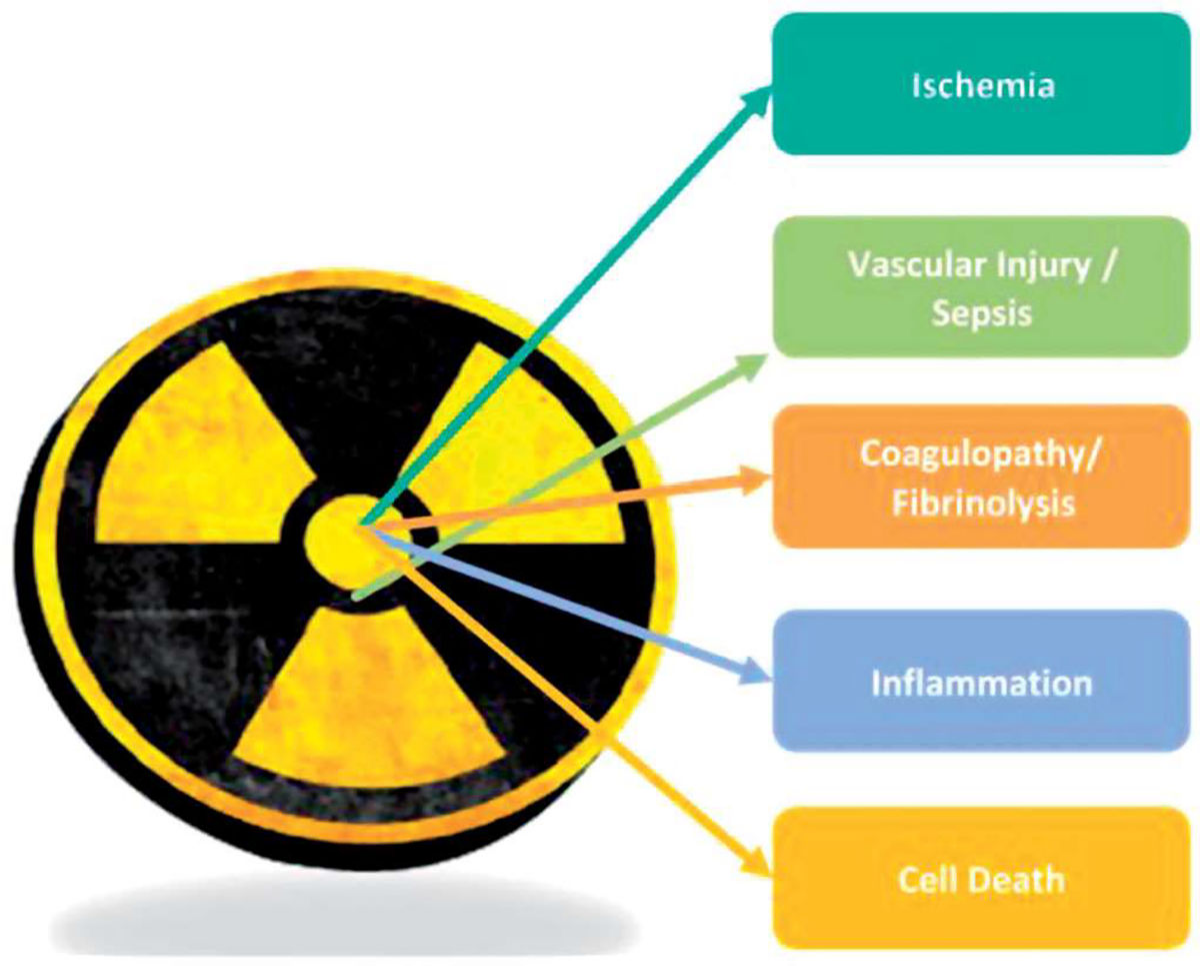
BARDA’s five focus areas for targeted product development indude vascular injury, coagulopathies, inflammation, cell death, and ischemla.

Dr. Mary Homer (Homer et al. 2016), BARDA, gave a talk on their ‘Radiological and Nuclear Countermeasure Program’, addressing areas of focus for preparedness in order to treat injury due to exposure of acute ionizing radiation caused by improvised nuclear device (IND) or radiological dispersal device (RDD) events with priority given to IND-related injuries since the impact is predicted to be greater. BARDA focuses on MCM candidates that are ready for advanced development. Due to the complex spectrum of injuries that are anticipated to include combined injuries of acute radiation exposure, trauma, and thermal burn, treatment is expected to require a polypharmacy approach (Yoo et al. 2014; Singh et al. 2019). Over the years, BARDA has evolved its focus away from organ-centric syndromes to focus on more pathophysiological processes involved in radiation injury. The five focus areas for targeted product development include: vascular injury, coagulopathies, inflammation, celi death, and ischemia (PHEMCE 2017–2018) (Figure 4). In the near term, the primary MCM development areas are for treatment of hematopoietic injury, specifically targeting thrombocytopenia and vascular injury.

Dr. Lynne Wathen, BARDA, gave a brief presentation on the development of radiation biodosimetry tests that may be useful during space missions or a mass casualty incident on earth. Biodosimetry is the measurement of physiological, Chemical or biological markers of exposure of human tissues to ionizing radiation. It offers an added dinical benefit to patient observation for post-irradiation symptoms by estimating qualitative and quantitative absorbed ionizing radiation dose. A point-of-care (POC), immediate qualitative test can deliver dose prediction to triage low- and no-absorption victims ffom all others. In addition, a quantitative low- or no-exposure test delivered quiddy can inform physicians in advance of diagnòstic neutropenia and the onset of acute radiation syndrome (ARS). Further, it can substitute a less efficient empirical treatment regimen with better-informed therapeutic management and consequently better allocation of scarce MCM resources. These two types of tests are currently under development with support ffom the United States Department of HHS (Larsen and Disbrow 2017). Initial assessments of test accuracy and positive/negative predictive values over a range of 0–10 Gy are underway using extensive dinical and non-dinical validation studies (Clinical Trials.gov 2017; Park et al. 2017; Jacobs et al. 2018).

### National Institutes of Health/National Heart Lung and Blood Institute

Dr. Keith Hoots, NHLBI, gave a presentation on vascular injury and the pathogenesis of endothelial injury. Chronic radiation exposure and its effect on the vascular cell repair machinery was a focus area along with determining if there is an impact of low, chronic radiation exposure due to cross-talk between the endothelium and circulating inflammatory celis. Another area of common interest includes CNS implications for chronic low-dose radiation exposure since key endothelial celi regulatory receptor activation appears to be relevant to inflammatory signaling across the blood-brain barrier (Kim et al. 2014; Venkatesulu et al. 2018). Long-term radiation exposure and the impact on long non-coding RNAs in the vascular endothelium and other human celis was a key topic discussed. NASA and NHLBI share areas of research interest in understanding the effect of chronic, low-dose radiation on the vascular system along with the mechanisms underlying the impact and the relationship of these events to the CNS.

### National Aeronautics and Space Administration (NASA)

NASA representative, Dr. Lisa Carnell, gave an overview of the risks ffom exposure to Space Radiation that may require physical and/or MCMs. Four primary risk areas were discussed including, Acute Radiation Sickness, Cancer, Degenerative Tissue and Central Nervous System Effects, each with multiple endpoints that intersect with the various agencies and institutes in different areas.

NASA has to address two different radiation problems on long-duration deep space missions, SPEs and GCR. Each needs to be addressed individually. In the case of SPE, there is the potential for prodromal and H-ARS effects at doses < lGy. Mitigation strategies include: (1) storm shelters with active dosimetry; (2) space weather forecasting and operations scheduling that reduce exposure during extravehicular activities and provide notification for crew to shelter; and (3) MCMs that may include treatments for nausea and vomiting along with G-CSF, Peg-G-CSF or GM-CSF for H-ARS (Kennedy 2014; Carnell et al. 2016), depending on the mission scenario.

Galactic cosmic radiation is the second radiation problem for NASA to address. GCR is comprised of approximately 87% protons (hydrogen nuclei), 12% helium nuclei, with 1% being the nuclei of heavier elements, called HZE ions (Simpson 1983). GCR is an even greater challenge because there are multiple effects to consider including the risk of CNSs disorders, and degenerative tissue effects in-flight, and late effects that may include the CNS, cardiovascular and other degenerative tissues along with solid and hematological cancers (Boerma et al. 2015; Huff et al. 2016; Nelson et al. 2016). Identifying an MCM to address multiple indications is challenging. NASA has determined that an ideal MCM will provide cross risk mitigation by targeting common pathways for each health impact. An ideal MCM to address GCR is defined in Table 1 (Carnell 2019). Requirements for including an MCM in the medical kit to address radiation-induced health effects will depend on the mission scenario. A key aspect for MCM consideration by NASA on long-duration missions is storage and shelf-life. A lyophilized form of MCM may provide longer stability and weight savings.

NASA has several areas of complementary interests with each of the agencies identified beyond what was highlighted already. There is a common need for extended shelf-life and storage for NASA and BARDA due to the need to include MCMs in the SNS. NASA has a demographic aligned with DOD since the astronaut corps is highly trained and monitored similar to the military, while many of the other agencies are addressing the general population. Determining the exposure dose is of concern to all agencies, as is developing computational modeling scenarios to predict the risk of exposure resulting in adverse health effects to the public and astronauts.

## Summary

Several federal agencies and institutes including NASA, NIH/NIAID, NIH/NCI, DoD/DTRA, NIH/NHLBI and ASPR/BARDA presented their areas of research at an interagency panel session held at the NASA HRP IWS on 26 January 2017. While the primary purpose of the panel session was to learn about the different focus areas of research conducted by each agency to determine if NASA could leverage partnership support, it became clear that there were areas of synergy that would be mutually beneficial across many of the agencies. Development and testing of MCMs in response to potential anti-terrorism activities that may involve weapons of mass destruction, dirty bombs or other means of radiation exposure was the focus for most of the agencies that presented. Although this research did not directly aligned with NASA’s needs, particularly since the exposures studied are acute, high doses of radiation; some cases of interest to both parties included a mixed field of gamma and neutrons, though at much lower doses and dose-rates for NASA’s interest. NASA representatives discovered they could learn a great deal from their agency and institute partners, particularly when addressing the possibility of H-ARS ffom SPE. Interagency collaborations have begun to form due to several other complementary areas of research identified during this panel session. Another common area of interest is in low-dose, chronic radiation exposures and the impact on the vascular and microvascular system. This resulted in an interagency agreement with BARDA to collaborate with NASA on a joint project to apply the NASA VESsel GENeration Analysis (VESGEN) software as an analysis tool of vascular patterning to quantify changes in the microvasculature post-radiation exposure. The interagency panel session also led to a joint workshop with NIAID on ‘Neutrón Radiobiology and Dosimetry’ held in the spring of 2019. Additional activities are currently in the planning stages. These collaborative efforts will help expedite research and maximize cost savings for all agencies involved.

## Figures and Tables

**Table 1.** Medical countermeasure criteria for GCR radioprotection/mitigation.

Medical products and regimens that prevent and/or mitigate adverse health effects due to space radiation with emphasis on broad activity (i.e. multi-tissue)
Mechanism of action well known
Independent of sex
Capable of being delivered chronically for the period of the mission (potentially up to 3 years)
Easily administered; capable of self-administration (e.g. oral, inhaled)
No contraindications with other drugs used for treating other symptoms or diseases during the mission
Known/potential benefits greater than known potential risks; minimal adverse events
Long shelf-life

## Abbreviations:

AFRRI: Armed Forces Radiobiology Research Institute
ARS: acute radiation syndrome
ASPR: Assistant Secretary for Preparedness and Response
BARDA: Biomedical Advanced Research and Development Authority
CMCR: Center for Medical Countermeasure Research
CNS: central nervous system
DEARE: delayed effects from acute radiation exposure
DTRA: Defense Threat Reduction Agency
DOD: Department of Defense
FDA: Food and Drug Administration
GCR: galactic cosmic radiation
Gl: gastrointestinal
Gy: Gray
HRP: Human Research Program
IND: improvised nuclear device
IWS: Investigator’s Workshop
ISS: International Space Station
MCM: medical countermeasure
NASA: National Aeronàutics and Space Administration
NCI: National Cancer Institute
NHLBI: National Heart Lung and Blood Institute
NIAID: National Institute for Allergy and Infectious Disease
NIH: National Institutes of Health
PDSS: Product Development Support Services
PHEMCE: Public Health Emergency Medical Countermeasures Enterprise
POC: point-ofcare
RDD: radiological dispersal device
RNCP: Radiation Nuclear Countermeasure Program
RRP: Radiation Research Program
SBIR: Small Business Innovation Research
SNS: Strategic National Stockpile
SRPE: Space Radiation Program Element
VESGEN: VESsel GENeration Analysis
